# CAR T cells in solid tumors: challenges and opportunities

**DOI:** 10.1186/s13287-020-02128-1

**Published:** 2021-01-25

**Authors:** Faroogh Marofi, Roza Motavalli, Vladimir A. Safonov, Lakshmi Thangavelu, Alexei Valerievich Yumashev, Markov Alexander, Navid Shomali, Max Stanley Chartrand, Yashwant Pathak, Mostafa Jarahian, Sepideh Izadi, Ali Hassanzadeh, Naghmeh Shirafkan, Safa Tahmasebi, Farhad Motavalli Khiavi

**Affiliations:** 1 Department of Hematology, Faculty of Medicine, Tabriz University of Medical Sciences, Tabriz, Iran; 2 Stem Cell Research Center, Tabriz University of Medical Sciences, Tabriz, Iran; 3 Kidney Research Center, Tabriz University of Medical Sciences, Tabriz, Iran; 4 The Laboratory of Biogeochemistry and Environment, Vernadsky Institute of Geochemistry and Analytical Chemistry of Russian Academy of Sciences, Kosygina 19 Street, Moscow, Russian Federation 119991; 5 Department of Pharmacology, Saveetha Dental College and Hospital, Saveetha Institute of Medical and Technical Sciences, Saveetha University, Chennai, India; 6 Department of Prosthetic Dentistry, First Moscow State Medical University, Moscow, Russian Federation; 7 Tyumen State Medical University, Tyumen Industrial University, Tyumen, Russian Federation; 8 Toxicology and Chemotherapy Unit (G401), German Cancer Research Center, 69120 Heidelberg, Germany; 9 DigiCare Behavioral Research, Casa Grande, AZ USA; 10 Taneja College of Pharmacy, University of South Florida, Tampa, FL USA; 11 Department of Immunology, Faculty of Medicine, Tabriz University of Medical Sciences, Tabriz, Iran; 12 Department of Virology, Pasteur Institute of Iran, Tehran, Iran

**Keywords:** Chimeric antigen receptor, Solid tumors, CAR T cells, Cell therapy

## Abstract

**Background:**

CARs are simulated receptors containing an extracellular single-chain variable fragment (scFv), a transmembrane domain, as well as an intracellular region of immunoreceptor tyrosine-based activation motifs (ITAMs) in association with a co-stimulatory signal.

**Main body:**

Chimeric antigen receptor (CAR) T cells are genetically engineered T cells to express a receptor for the recognition of the particular surface marker that has given rise to advances in the treatment of blood disorders. The CAR T cells obtain supra-physiological properties and conduct as “living drugs” presenting both immediate and steady effects after expression in T cells surface. But, their efficacy in solid tumor treatment has not yet been supported. The pivotal challenges in the field of solid tumor CAR T cell therapy can be summarized in three major parts: recognition, trafficking, and surviving in the tumor. On the other hand, the immunosuppressive tumor microenvironment (TME) interferes with T cell activity in terms of differentiation and exhaustion, and as a result of the combined use of CARs and checkpoint blockade, as well as the suppression of other inhibitor factors in the microenvironment, very promising results were obtained from the reduction of T cell exhaustion.

**Conclusion:**

Nowadays, identifying and defeating the mechanisms associated with CAR T cell dysfunction is crucial to establish CAR T cells that can proliferate and lyse tumor cells severely. In this review, we discuss the CAR signaling and efficacy T in solid tumors and evaluate the most significant barriers in this process and describe the most novel therapeutic methods aiming to the acquirement of the promising therapeutic outcome in non-hematologic malignancies.

## Introduction

**Fig. 1:**
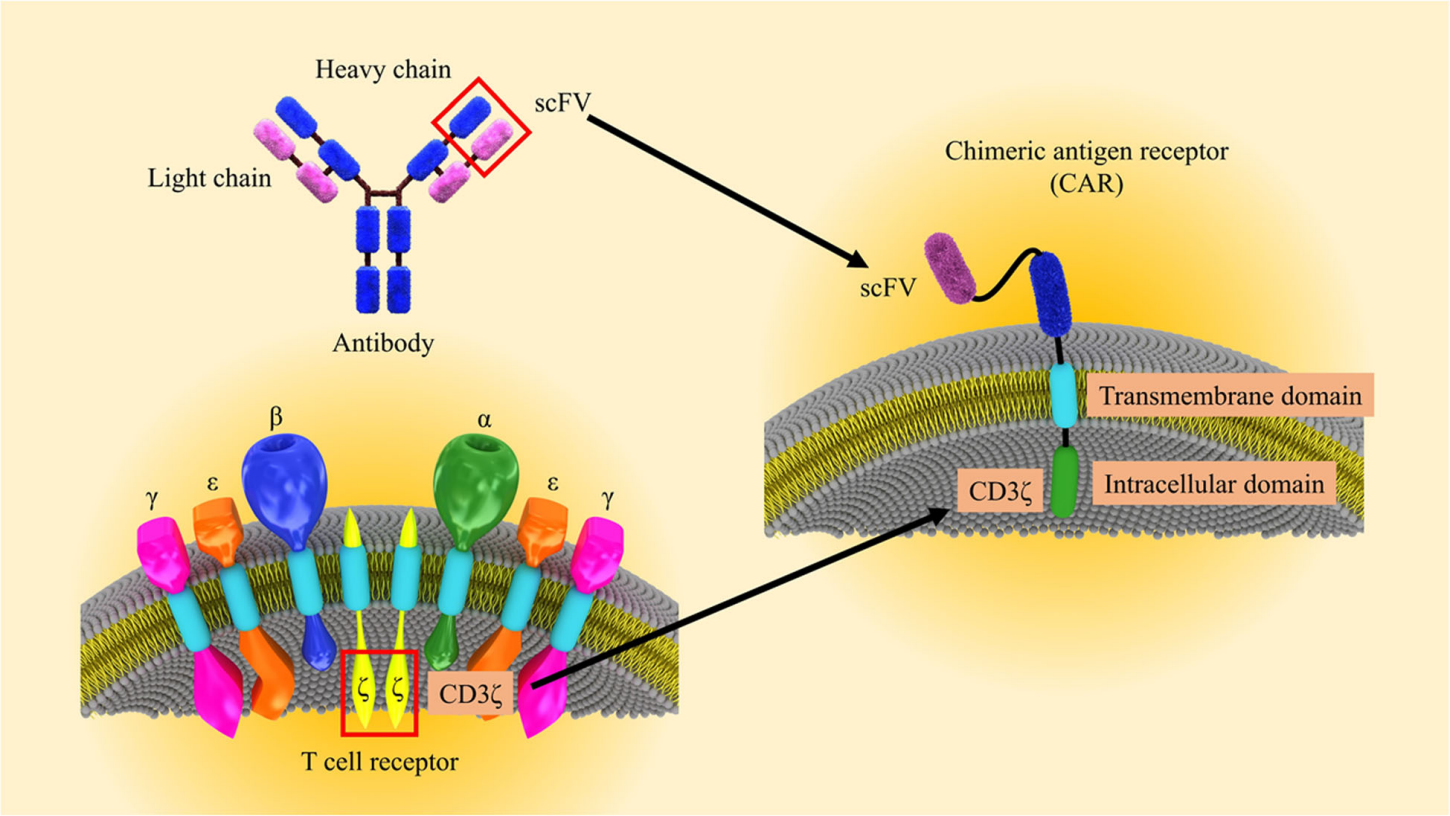
CAR T cell engineering. The design of the CAR T cell has evolved by combining existing immune cell components to facilitate direct targeting of tumor antigens. scFv of CAR-induced light and heavy chains of the antibody variable region, whereas the CAR amplitude CD3ζ has been derived from the intracellular signaling domains of the TCR

Chimeric antigen receptor (CAR) T cell therapy signifies an attractive cellular immunotherapy tactic to cancer treatment that takes the benefit of exclusive properties of the T cells [1]. CARs are recombinant receptors for cell surface antigen redirected the specificity and activity of blood T lymphocytes and other types of the immune cells [2]. The rapid establishment of tumor-targeted T cells, elimination of obstacles, and augmentative kinetics of active immunization are noticed as the general principle of CAR T cell application in cancer. The CAR-modified T cells obtain supra-physiological possessions and performance as “living drugs” that could demonstrate not only immediate but also long-term effects following expression in T cells [3]. For CAR engineering in T cells, the cells must be cultured so that they can be used for transduction and expansion. In this process, the transduction may exploit a diversity of approaches, but established gene transfer is obligatory to enable persistent CAR expression in the clonally expanding and persevering T cells (Fig. 1) [4]. Considering the principles, an antigen expressed on the cell surface can be targeted by a CAR and varied T cell subsets, T cell progenitors, and other immune cells, in particular, natural killer (NK) cells, can be targeted by a CAR [5]. The establishment of the immune reactivity against special antigen is not the only therapeutic goal of smarter CARs, and these cells are designed to achieve much more than to trigger engineered T cell activation and functions. Importantly, CARs with notable potential and signaling quality can regulate T cell expansion and perseverance, and the strength of engineered T cell activation in the cancer microenvironment, properties that intensely modify cancer-targeted T cell efficacy together safety. Based on the biological and molecular investigations, CAR delivery has a wider spectrum of functional effects than transduced T cell receptors (TCRs), in which power of signaling that is generally modified by the affinity of TCRs to the target antigen is the central factor in the determination of T cell fate [6]. Although flexibility is associated with the dynamic range of the engineered CARs and is very promising and ideal, CARs are restricted to identify markers sited on the cell surface. On the other hand, CARs induce cell death in target cells without any dependency on the MHC molecules [7]. We here argue targeting and signaling possessions of the engineered CARs, considering their impacts on T cell specificity in association with potency as well as safety. Furthermore, the procedures involved in T cell expansion and this cell subset collection are discussed in this review. Overall, based on the modular nature of chimeric antigen receptor construction, CARs are swiftly developing and demonstrate a remarkable capacity for their effective use in a wide spectrum of immunotherapies [8].

## CAR structure

**Fig. 2:**
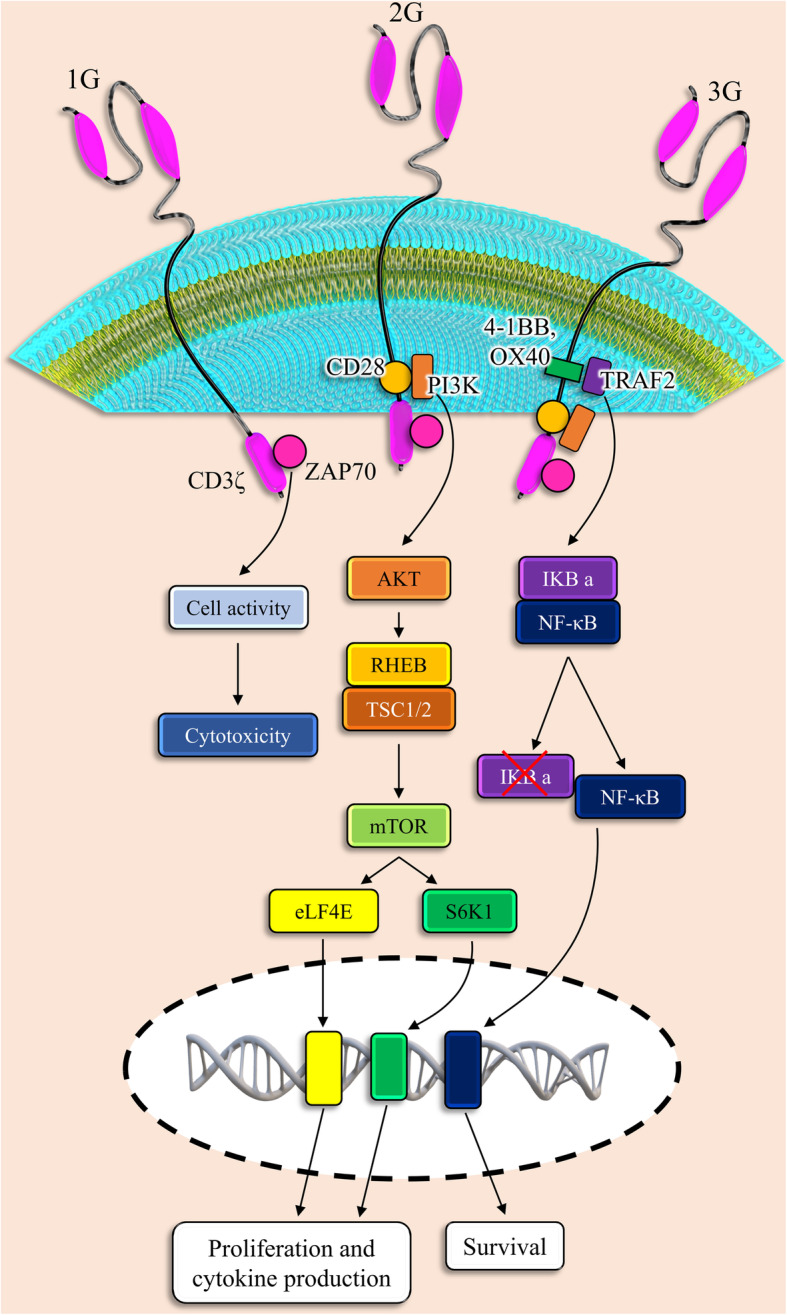
CAR T cell engineering. The specificity of T cells against tumor cells is mediated by CAR proteins. CAR is a combination of extracellular proteins and is usually derived from antibodies and intracellular signaling modules derived from T cell signaling proteins. First-generation CARs have been composed of CD3ζ, while adding  a costimulatory endodomain including CD28 or 4BB to CD3ζ has led to the creation of second-generation CARs. Third-generation cars include two costimulatory domains fused to CD3ζ. VH, variable heavy chain; VL, variable light chain; scFv, single-chain variable fragment. Generation of CAR T cells leads to the initiation of different signaling pathways which caused cell survival, proliferation, and cytokine production

CARs contain an extracellular antigen-identifying domain, which is constructed by fragments of monoclonal antibodies identifying a particular protein on the cell membrane of the cancerous cells (e.g., EGFR on solid tumor cells or CD19 on B cells) and an intracellular stimulating domain that provides the T-cell receptor (TCR) signaling to trigger CAR T-cell activation and function [9, 10]. First-generation CAR T-cells contained an intracellular domain from the TCR CD3 ζ-chain that induced T cell cytotoxicity effect against targeted cancer cells but could not to promote CAR T cell expansion in vivo following reinfusion; on the other hand, second- and third-generation CAR T cells contained additional co-stimulatory intracellular domains, which in turn augmented the CAR T cells’ potential to grow, expand, and finally be persistent in the patient’s body (Fig. 2) [11–13].

## CAR T cell engineering

CARs began to be studied in various ways when protocols for the transduction of human primary T cells were developed. In the last decade, almost all CAR studies have been based on using retroviral vectors, such as gamma-retroviral and also lentiviral vectors [14]. While retroviral vectors are able to stimulate insertional oncogenesis in human cells, T cells seem to be far less sensitive to these transformations. Transposases, which support arbitrary vector integration, are beginning to be assessed in the field of CAR therapy [15]. Although the advantages/disadvantages of commonly used vectors have not yet been clarified, this proportion has a tight association with CAR expression ranges, silencing over time, engineering easiness, safety properties, etc. However, T cell transformation due to the insertional mutagenesis has not been confirmed to date; direct integration of the vector into the safe regions of the genome is able to finally lead to the long-term CAR expression without insertional mutagenesis occurrence risk [16]. Alternative tactics independent of transgene integration, which used either RNA electroporation or cell surface conjugation, in turn, lead to the transient CAR expression and limitation of CAR T cell persistence beyond 7–14 days [3]. The notable properties of transiently CAR-expressing T cells that likely require several infusions to prepare acceptable tumor responses may attenuate normal tissue damage or avert T cell gathering to levels supporting the risk of cytokine storms, residues to be established. In this regard, another important aspect of CAR transfer is the addressee and recognizing what types of T cells (e.g., CD4+, CD8+ αβ T cells, and γδ T cells) are superior to other cells with the aim of optimal tumor suppression [17, 18].

## Intracellular signaling pathways of the CARs

As noticed, the first engineered receptors that presented noticeable T cell-stimulating ability were chimeric molecules between CD3-ζ or Fc receptor γ and CD8, CD4, CD25, or CD16, which stimulated the phosphatidylinositol and tyrosine kinase signaling cascades associated with calcium influx in the human leukemic T cells [19, 20]. A murine antibody hapten-specific scFv was added to the extracellular parts of these fusions, which were described as T-body and acceptably promoted cytolysis. Although CD3-ζ chain accretion is adequate to support cytolytic function in cytotoxic T cells (CTL) lines, it has been found that the strength of the essential signal to the presentation of the cytotoxic activity is lower compared with the other types of their functions [21]. This possibility highlights the restricted beneficial reactions demonstrated with activating receptors, the anti-cancer properties of which are commonly limited to not only models with non-systematic administration but also short-term systemic models. Considering studies, engineered CAR T cells that merely include stimulation domain in their cytoplasmic parts are susceptible to showing anergy in transgenic mice [22]. Once researchers could proficiently transduce human primary T cells, they noticed that CD3-ζ CARs could not stimulate the vigorous release of cytokine and improve T cell growth after identifying the target antigen. Thus, they tried to design novel types of the chimeric receptor, owning both stimulatory and costimulatory possessions, to support the robust expansion of T cells after identifying the target antigen. Consequently, second-generation CARs, containing the CD3-ζ chain in association with the costimulatory receptor cytoplasmic domain (e.g., CD28 and 4-1BB), were designed. The desirable functions of the second generation of CARs compared with the first generation of CARs were shown in varied types of models using the mouse or human T cells [23–25]. The main property of dual-signaling receptors is to support the superior potential of signaling and perseverance to the T cells, ensuing in these cells’ general superior potency. The improved perseverance established by the second generation of CARs has been approved in individuals exposed to CAR T cells with either a CD28/CD3ζ or CD3ζ-only CAR [26]. Although the second-generation CARs are designed in a different configuration, there not exist meticulous comparisons. The efficiency of some second-generation CARs based on CD28 and 4-1BB was examined in animal models; however, either one was shown to be more effective than others in various circumstances. In one investigation, although researchers found no significant differences in therapeutic activity of CD28- and 4-1BB-based CD19-specific CARs, they described that the T cells expressing the CD19-BB CAR gathered to superior levels, probably in the antigen-independent manner [27]; on the other hand, cited difference was not approved in other models [28]. It seems that more comprehensive studies are required, noticing that these studies must focus on the differences between chimer receptors within any one given class. For example, various CD28/CD3ζ CARs vary in their potential to prompt interleukin-2 secretions [29, 30]. Moreover, targeted epitopes’ special positions, their concentration in association with the CARs affinity, and other topological impacts of CAR structure could modify CAR signaling. The third generation of CARs, containing two different costimulatory domains collective with a special activation domain in their cytoplasmic section, demonstrated a greater ability to the treatment of solid tumors in several mouse models [31, 32]. While the first clinical research using CD20-specific CD28/4-1BB/CD3ζ did not expose desirable responses, these results should not diminish from the therapeutic importance of these “triple-decker” chimeric receptors [33]. In total, more study is wanted to acquire a more comprehensive understanding of optimal CAR signaling to improve persistent T cell activity and viability, declining premature death rate, swift exhaustion, or uncontrolled progression.

## Recognition of the tumor-associated antigens, expression level, and susceptibility to CAR T cells

**Fig. 3:**
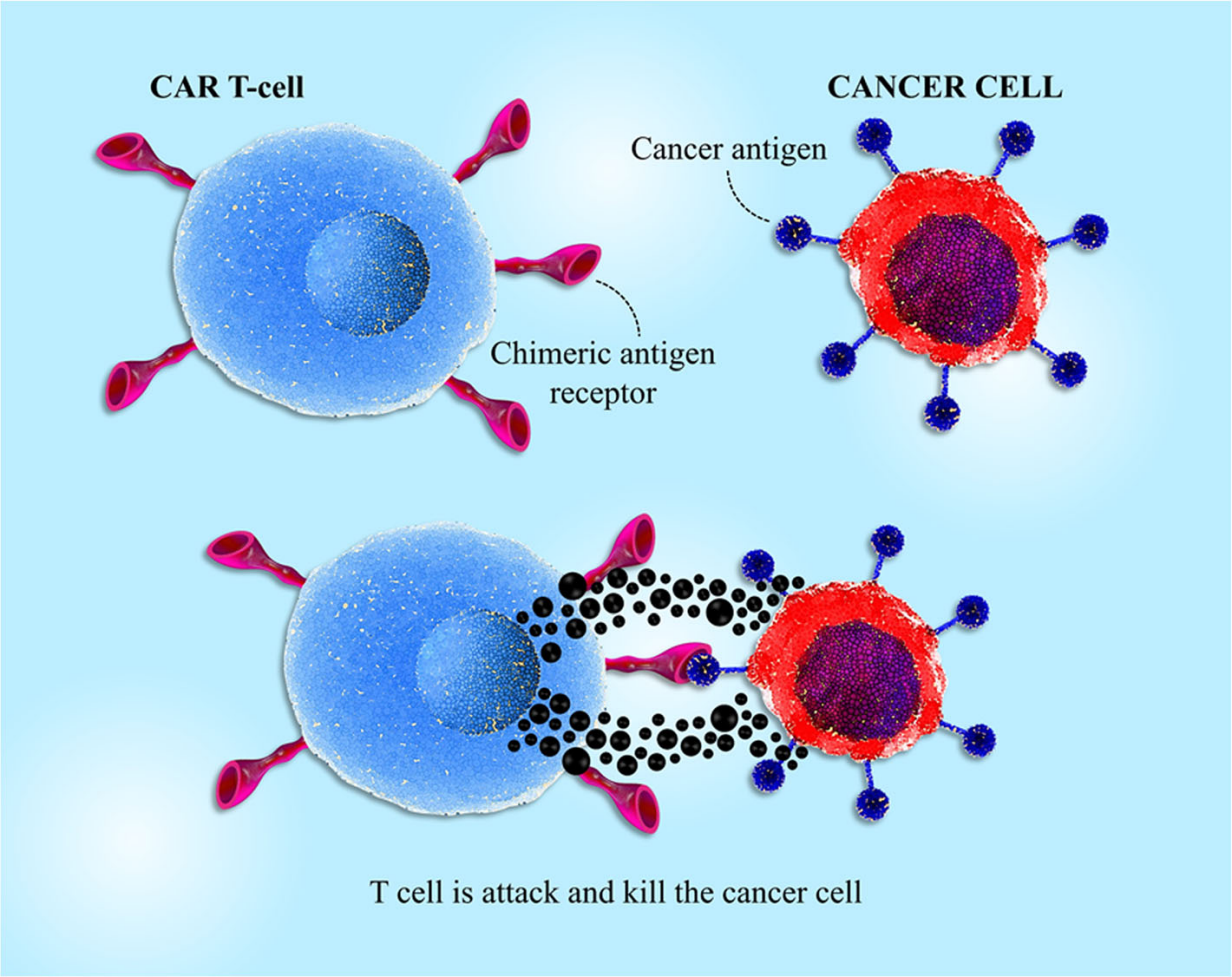
T cell-mediated antitumor effects by chimeric antigen receptors. CAR-modified T cells can detect tumor cells by CAR binding to TAA independent of the TCR-MHC/peptide interaction

The chief dissimilarity between solid tumors and blood disorders is that it is further intricate to detect a perfect target antigen (Fig. 3). Unlike hematological malignancies in which the cancer cells commonly express the special and individual markers, solid tumors often do not express one tumor-specific marker. In solid tumors, usually, it is more common to recognize a tumor-associated antigen (TAA) wherever the expression of markers, such as CEA, ERBB2, EGFR, GD2, mesothelin, MUC1, and PSMA, is increased on cancer cells. It should be noted that these markers are also expressed at a low degree on the human body’s natural tissues [34, 35]. Undoubtedly, in the absence of the tumor antigen specificity, the risk of significant on-target off-tumor toxicity is remarkably augmented. This catastrophic toxicity occurred for a patient with metastatic CRC who received Her2-CAR T cells [36] and a neuroblastoma patient who was treated with GD2-CAR T cells [37]. These disappointing events highlight the value of identifying a safe TAA since significant toxicity can ensue even from the lower rate of the special antigen, according to reports. Moreover, these reactions also indicated that there is a close association between the connecting affinity of a CAR and its related safety and efficacy. An in vivo research showed that utilizing ICAM-1-specific CAR T cells with μM affinity had a low-level side effect and was more effective compared with CARs with nM affinity [38, 39]. Besides, studies demonstrated that CAR with lower affinity exhibited low-level exhaustion and promoted proliferation in vivo. In this regard, other studies demonstrated that GUCY2C-specific CAR T cells, a receptor expressed in about 95% of metastatic CRC were safe and effective in not only immunocompetent mice with aggressive cancer but also in human xenograft models [40]. In total, aberrantly or overexpressed antigens on tumors expressed on normal tissues must be carefully assessed to be described as a target antigen for solid tumor therapy. In the last decade, various experimental groups exploited immunoproteomics to recognize TAAs utilizing autoantibodies toward immunogenic antigens that are functionally expressed either in the cytosol or on the surface of cancerous cells [41]. These target antigens may be completely unrecognized proteins, well known as neoantigens, or wild-type mutated peptides entitled as neoepitopes [42]. PSMA1, LAP3, ANXA3, and maspin are some of the TAAs recognized using proteomics that are considered as biomarkers for CRC [43]. The neoantigens can also be recognized through DNA or RNA sequencing and also using whole-exome screening to investigate somatic mutations in cancers [44, 45]. Investigations based on using whole-exome sequencing of melanoma [46] and glioblastoma multiforme (GBM) samples exhibited multiple mutated epitopes in these patients [42]. For neoantigen prediction, whole-exome sequencing was conducted in the PDA patients, and it has been found that more numbers of neoantigens in association with more numbers of CD8+TILs support promoted survival [47]. Some studies have assessed CD40 agonist’s potential to improve the immunity of T cell to solid tumors and found that CD40 agonists are able to boost T cell response to dimly immunogenic tumor antigens [48]. In this regard, in PDA models, combining chemotherapy with CD40 agonists, presented the infiltration process of T cell and neoantigen-specific response and tumor suppression [49]. These investigations using neoepitopes exhibit that tumors can trigger secondary immune responses toward previously unidentified markers and that neoantigen-related endogenous immunity possibly adjusts tumor progression. These findings highlight the importance of adoptive T cell therapy such as CAR-based therapeutic approaches. While a large number of studies confirm neoepitopes’ potential to recognize pre-existing TCR reactivity, detection of neoepitopes and exploiting of CAR T cells to target these epitopes could supposedly detour the significance of this subject as CARs act as an MHC-independent receptor.

As cited, solid tumors incline to show a great level of antigen heterogeneity. According to studies, tumors usually have only cell divisions that strongly express the target antigen, and there is usually a risk of the target antigen being destroyed and removed from the cancer cells [50].

Although this event has already been demonstrated with leukemia cells after transfusion of CD19-CAR T cell, the involved process is not well identified [51]. However, one study investigates a particular mutation caused in a form of CD19, which lost the special epitope targeted by the CD19-based CAR T cells [52]. In the clinical trial using EGFRvIII-specific CAR for the treatment of GBM, CAR T cell administration led to the inhibition of the EGFR/EGFRvIII receptor expression and seemed to reinforce T cell resistance, but infusion was displayed to be non-toxic and also effective [53]. Moreover, in the GBM model, a CAR T cell-based IL13Rα2 expanded and released various cytokine in vivo, but inhibition of IL13Rα2 expression was shown in recurrent tumors [54].

## CAR T cell therapy in solid tumors: recent advances

**Fig. 4:**
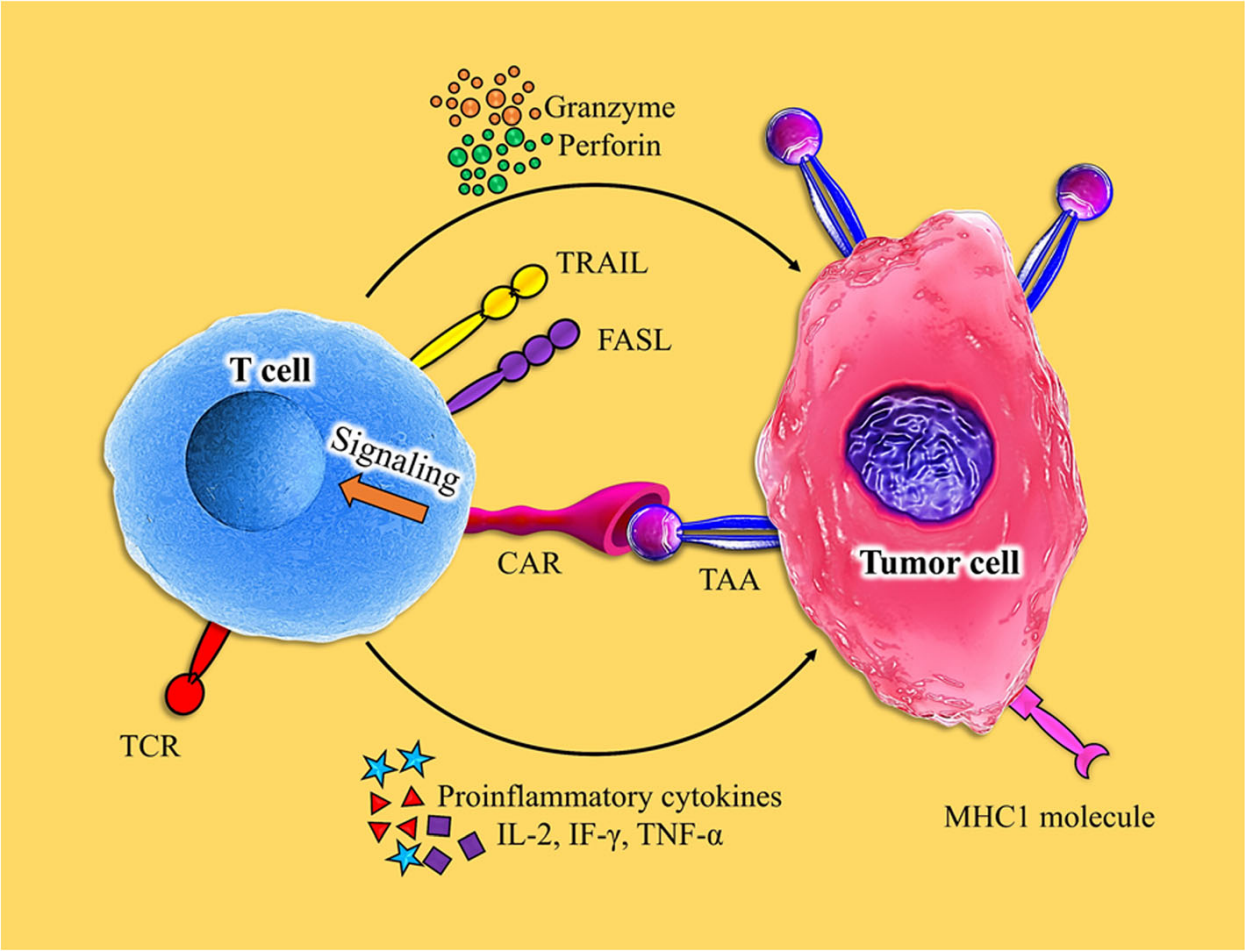
T cell-mediated antitumor effects by chimeric antigen receptors. T cells are activated and can kill tumor cells by secreting granzymes and perforin, as well as the expression of TRAIL and FasL. Moreover, immune cells that invade the tumor can be activated by secreting various cytokines

Given the numerous methods enabling tumors to suppress T cells, the number of cell engineering and combination therapies, which can be examined in the clinic, is infinite. In this regard, authentic preclinical models to scrutinize therapeutic combinations are highly interesting before clinical translation. Although our focus is not on CAR-based trials, in this part, we evaluate recent studies in solid tumor CAR T therapy and discuss their efficacy and important targeted surface markers, briefly (Fig. 4) (Tables 1 and 2).

**Table 1:** Most targeted antigen in clinical trials in solid tumor CAR T cell therapy

Antigen	Cancer	Phase	ID
EGFR	Lung, liver, stomach	Phase 1/2	NCT03179007, NCT03525782
HER2	Central nervous system tumor, pediatric glioma	Phase 1	NCT03500991
EGFR806	Central nervous system tumor, pediatric glioma	Phase 1	NCT03179012
Mesothelin	Ovarian, cervical, pancreatic, lung	Phase 1/2	NCT01583686
PSCA	Lung	Phase 1	NCT03198052
MUC1	Advanced solid tumors, lung	Phase 1/2	NCT03179007, NCT03525782
Claudin 18.2	Advanced solid tumor	Phase 1	NCT03874897
EpCAM	Colon, pancreatic, prostate, gastric, liver	Phase 1/2	NCT03013712
GD2	Brain	Phase 1	NCT04099797
VEGFR2	Melanoma, brain	Phase 1	NCT01218867
AFP	Hepatocellular carcinoma liver cancer	Phase 1	NCT03349255
Nectin4/FAP	Nectin4-positive advanced malignant solid tumor	Phase 1	NCT03932565
CEA	Lung, colorectal, gastric, breast, pancreatic cancer	Phase 1	NCT02349724
Lewis Y	Advanced cancer	Phase 1	NCT03851146
Glypican-3	Liver	Phase 1	NCT02932956
EGFRIII	Glioblastoma and brain tumor	Phase 1	NCT01454596
IL-13Rα2	Glioblastoma	Phase 1	NCT02208362
CD171	Neuroblastoma	Phase 1	NCT02311621
MUC16	Ovarian	Phase 1	NCT02311621
PSMA	Prostate	Phase 1	NCT01140373
AFP	Hepatocellular carcinoma, liver	Phase 1	NCT03349255
AXL	Renal	Phase 1	NCT03393936
CD20	Melanoma	Phase 1	NCT03893019
CD80/86	Lung	Phase 1	NCT03198052
c-MET	Breast, hepatocellular	Phase 1	NCT03060356, NCT03638206
DLL-3	Lung	Phase 1	NCT03392064
DR5	Hepatoma	Phase 1	NCT03638206
EpHA2	Glioma	Phase 1	NCT02575261
FR-α	Ovarian	Phase 1	NCT00019136
gp100	Melanoma	Phase 1	NCT03649529
MAGE-A1/3/4	Lung	Phase 1	NCT03356808, NCT03535246
LMP1	Nasopharyngeal	Phase 1	NCT02980315

*EGFR* epidermal growth factor receptor, *HER2* human epidermal growth factor receptor 2, *PSCA* prostate stem cell antigen, *MUC1* mucin1, *EpCAM* epithelial cell adhesion molecule, *AFP* alpha-fetoprotein, *FAP* familial adenomatous polyposis, *CEA* carcinoembryonic antigen, *MUC16* mucin16, *PSMA* prostate-specific membrane antigen, *AXL* AXL receptor tyrosine kinase, *DLL3* delta-like 3, *EPHA2* EPH receptor A2, *FRα* folate receptor alpha, *LMP1* Epstein-Barr virus latent membrane protein 1, *MAGE* melanoma antigen gene protein, *DR5* death receptor 5

**Table 2:** Targeted antigens in solid tumor CAR T cell therapy (in vitro studies)

Cancer	Antigen
Colorectal	NKG2D [54], EP-CAM [55], HER2 [56], GUCY2C [57], TAG-72 [58], CD46 [58]
Liver	CEA [59], Glypican3 [60], AFP [61]
Gastric	Mesothelin [62], ANTXR1 [63], MUC3A [63], Trop2 [64], Claudin18.2 [66], NKG2D [28], HER2 [65, 66], FR-α [67]
Pancreatic	MUC1 [68], Mesothelin [69], αvβ6 [70], CEA [71], PSCA [71], FAP [71], CD47 [72], HER2 [73], NKG2D [74]
Renal	CAIX [75]
Melanoma	GD2 [76], GSPG4 [77], Glypican3 [78], HER2 [79]
Cervix	αvβ6 [80], L1-CAM [81]
Neuroblastoma	GD2 [82, 83], CD56 [84], Glypican 2 [85], CD171 [86]
Glioblastoma	EGFRvIII [87], HER2 [88], B7-H3 [89], NKG2D [90], CAIX [91], αvβ3, IL13Rα2 [92]
Ovarian	Mesothelin [62, 93], αvβ6 [94], B7-H3 [95], CD47 [72], NKG2D [96]
Prostate	PSA [97], PAP [97], PSCA [98], PSMA [99], EpCAM [100]
Lung	MAGE-A1 [101], CD32A [102], ROR [103], EGFRvIII [104]
Head and neck	HER2 [105]

*NKG2D* natural killer group 2, member D receptor, *EpCAM* epithelial cell adhesion molecule, *HER2* human epidermal growth factor receptor 2, *PSCA* prostate stem cell antigen, *MUC1* mucin1, *AFP* alpha-fetoprotein, *FAP* familial adenomatous polyposis, *CEA* carcinoembryonic antigen, *MUC16* mucin16, *PSMA* prostate-specific membrane antigen, *CAIX* carbonic anhydrase IX, *FRα* folate receptor alpha, *TAG-72* tumor-associated glycoprotein 72, *MAGE* melanoma antigen gene protein, *GUCY2C* guanylate cyclase 2C, *ANTXR1* anthrax toxin receptor 1, *GSPG4*; general secretion pathway protein G, *PSA* prostate-specific antigen, *ROR* RAR-related orphan receptors

### Ovarian cancer

Novel therapeutic methods for the treatment of ovarian cancer (OC) are immediately required due to its remarkable level of recurrence following surgery and multi-agent chemotherapy. Tumor-associated glycoprotein 72 (TAG72) expressed at a high rate on the surface of ovarian cancer has been used as a target of CAR-T cell therapy. According to reports, a humanized TAG72-specific CAR T cell demonstrated cytotoxicity potential and cytokine production in OC; on the other hand, TAG72-based CAR T cells meaningfully diminished proliferation potential and augmented experimented mice viability [55]. Other in vitro studies have revealed that MUC16-specific CAR T cells presented robust anti-tumor function in OC cells. It was found that intravenous or intraperitoneal injection of MUC16-CAR-T cells could decline ovarian cancer progression completely or eradicated malignant cells in mouse models. Investigations also approved the research importance of MUC16 as a potential target for ovarian cancer cell treatment [56]. On the other hand, studies presented that Her2-CAR-T cells were able to suppress the growth potential of the human ovarian SKOV3 cell line expressing Her-2/neu [56], and the use of the Meso-CAR-T cells [57] led to the inhibition of proliferation and promoted mice viability. Furthermore, 5T4-specific CAR T cells [106] and FRα-specific CAR T cells [58] demonstrated a noteworthy inhibitory effect on ovarian cancer cell growth and progression. In a recent study, CD19- and Mesothelin (MSLN)-CAR NK-92 cells were designed for the targeting of CD19 and MSN. The expression of both CD19- and MSLN-CAR molecules was significantly increased on the surface of NK-92 cells after lentiviral gene transfer. MSLN-CAR NK cells remarkably killed MSLN^+^ ovarian cancer cells including SK-OV-3 and OVCAR-3 in vitro [59].

### Breast cancer

Zhou et al. showed that after recognition of tMUC1 on triple-negative breast cancer (TNBC) cells, MUC28z CAR T cells, a specific composed chimeric antigen receptor containing the CD28 and CD3ζ domains, amplify the synthesis of Granzyme B, IFN-γ, and other types of cytokines and chemokines secreted by Th1. In this study, a single dose of MUC28z CAR T cells considerably decreased TNBC tumor proliferation and survival in a xenograft model [60]. Other research revealed that CD27 or 4-1BB costimulated, self-enriched NKG2D CAR-redirected T cells involved anti-cancer function toward TNBC tumor [61]. Other studies suggested that HRG1β-based CAR-T cells successfully inhibit breast cancer proliferation through HER family receptors and able to deliver an attractive therapeutic approach to defeat cancer resistance against HER2-based targeted therapy [62]. In parallel, Munisvaradass et al. found that human anti-HER2 CAR T cells showed desirable targeting and triggered cell death in HER2 overexpressing breast cancer cells [63]. Moreover, mesothelin recognition by special CAR T cells has been described as a promising immunotherapy goal for breast cancer treatment [64].

### Prostate cancer

Prostate stem cell antigen (PSCA) and prostate-specific membrane antigen (PSMA) are utilized commonly for targeting chimeric antigen receptors for reaching the appropriate therapeutic outcomes in prostate cancer (PC) [107]. The CAR T cells against PSMA show great ability toward human PC cells and demonstrated strong expansion as well as cytotoxicity potential in PC cells [28, 65]. Clinical trials conducted by Junghans et al. [66] and Slovin et al. [67] approved PSMA-directed CART cells’ safety and efficacy in PC.

### Renal cancer

Based on reports, carboxy-anhydrase-IX (CA-IX) expressed in varied types of renal cancers has been noticed as a novel target for CAR T cell therapy. CA-IX is a metalloprotease normally participating in the catalysis of carbon dioxide hydration [13, 68], whereas it is applicable as a critical antigen in renal cell carcinoma and several normal tissues, including gastric mucosa epithelium, small intestine epithelium, and duodenum, and biliary tree expresses it moderately [69]. Additionally, hypoxic conditions may lead to the CA-IX expression in wide ranges of tissues [70]. It has been revealed that first-generation CAIX-CAR T cells toward renal carcinoma cells participate in the secretion of the high degree of cytokine in association with cytotoxic function [71].

### Gastric cancer

Recent studies demonstrated that bi-specific Trop2/PD-L1 CAR-T cells can meaningfully decline gastric cancer growth through intratumoral injection, with a more prominent suppression effect than Trop2-specific CAR-T cells. These findings reveal that novel bi-specific Trop2/PD-L1 CAR-T cells participate in Trop2/PD-L1 and checkpoint blockade on gastric cancer, thereby promoting the cytotoxic effect of CAR-T cells in gastric and other types of solid tumors [72]. Besides, it has been verified that upon injection of mesothelin-CAR T cells encompassing the mesothelin scFv, CD3ζ, CD28, and DAP10 intracellular signaling domain (M28z10), gastric cancer cell death triggered and tumor growth remarkably inhibited [73]. Based on other studies, using claudin18.2-CAR T cells [74], NKG2D-CAR T cells [75], folate receptor 1 (FOLR1)-CAR T cells [76], and HER2-CAR T cells [77] can be considered as a novel therapeutic approach for gastric cancer therapy. In a recent study, Jung et al. showed that ICAM-1 CAR T cells alone or in combination with chemotherapeutic agent paclitaxel or CAR T cells modified IL-12 release, as a promising approach which greatly improves ICAM-1^high^-advanced gastric cancer patients [78].

### Pancreatic cancer

Studies verified that CXCR2-expressing CAR T cells transfer more powerfully toward IL-8 and IL-8 containing microenvironment in pancreatic cancers. As a result, CXCR2-expressing CAR T cells provoke greater anti-tumor activity toward recognized αvβ6-expressing pancreatic tumor xenografts [79]. Moreover, B7-H3.CAR-T cells’ efficacy in the treatment of pancreatic ductal adenocarcinoma in vitro and orthotopic as well as metastatic xenograft mouse models has been proven. Interestingly, 4-1BB co-stimulation supports lower PD-1 expression in generated T cells, and more antitumor activity when we want to target PD-L1 constitutively expressed tumor cells [80, 81].

Additionally, phase I clinical study on patients with hepatocellular carcinoma, pancreatic carcinomas, and colorectal carcinomas exhibited CD133-CAR T cell inhibitory effect on these cell metastasis potential [82]. Besides, other types of the target antigens for pancreatic cancer CAR T cell therapy, including CD24 [83], PSCA [84], CEA [85], MUC-1 [86], mesothelin [87], FAP [88], and Her-2 [89], have been known and are being investigated in preclinical and also clinical trials.

### Lung cancer

Treatment with receptor tyrosine kinase-like orphan receptor 1-specific (ROR1)-CAR T cells supports strong antitumor activity in human lung cancer A549 cell lines. Importantly, ROR1-CAR T cells infiltrate into cancerous tissue and eradicate multiple layers of tumor cells [90]. Similarily, EGFRvIII-CART specifically and proficiently identify and kill A549-EGFRvIII cells upon expressing and releasing cytokines, such as perforin, granzyme B, IFN-γ, and TNF-α; on the other hand, studies showed that the metastasis of A549-EGFRvIII cells in mice was robustly diminished by EGFRvIII-CART cells, and mouse survival was meaningfully extended without any side effects [91]. Also, it has been verified that CAR T cell-based mesothelin [92], erythropoietin-producing hepatocellular carcinoma A2 (EphA2) [93], and PSCA and mucin-1 [94] can lead to the desired therapeutic outcome in lung cancers. Recently, a group of researchers suggested that the use of PD-L1-CAR T in non-small cell lung carcinoma (NSCLC), potentially exhibited antitumor cytotoxic activity against PD-L1^high^ and EGFR^mut^ NSCLC and to some extent leads to the recovery of patients with (PD-L1^+^) NSCLC [95]. On the other hand, Chen and colleagues introduced delta-like 3 (DLL3) as an attractive target for the treatment of small cell lung cancer (SCLC). They showed that DLL3 targeted with antibody and CAR-T cell alone or along with PD-1 inhibition kill DLL3 tumor cells including H82, H196, and H446 cell lines [96].

### Liver cancer

The use of the CAR-T therapy for liver cancer treatment is just beginning to be investigated, and more studies are required. However, the potency of the CAR-T cell-based CEA [97], glypican-3 [98], mucin-1, epithelial cell adhesion molecule, and carcinoembryonic antigen [99] has been verified in liver cancer therapy. The use of Glypican-3 (GPC3) antibody in combination with CAR T therapy can be a useful method in the treatment of liver malignancies. Liu and coworkers showed that the use of 32A9 monoclonal antibody /CAR T cells kill (GPC3^+^) HCC cells in vitro and regresses liver xenograft tumor in vivo [100]. Another study illustrated that GPC3/CAR T cells expressing IL15/21 promoted the antitumor responses of T cells against HCC [101].

### . Colorectal cancer

Chimeric antigen receptor T cell therapy may be an effective treatment method for colorectal cancer, according to findings. Overall, in colorectal cancer, NKG2D [102], CD133 [82], GUCY2C (Guanylate Cyclase 2C) [40], and TAG-72 [103] are the most prominent target antigen to reaching the promising therapeutic goal. Humbach et al. showed that mesenchymal stem cells (MSCs) engineered to release IL-7/12 cytokines increase the anti-tumor activity of CAR T cells against colorectal carcinoma cells by altering the inflammatory action of Th2 to Th1/17 executive profile in the tumor milieu [104]. According to previous evidence, raised levels of Doublecortin-like kinase 1 (DCLK1) expression in human colorectal tumors are associated with higher mortality rates. A recent report revealed that DCLK1’s targeted CAR-T therapy effectively eradicates primary and metastatic colon cancer cells [105].

## Challenges of CAR T cell therapy for solid tumor

This section discusses the fundamental challenges of CAR T cell therapy in solid tumors as well as useful strategies to enhance the therapeutic effects. The challenges listed below are the most important barriers to interfering with cell therapy and affect the usefulness of treatment depending on the type of tumor, the step of the disease, and the molecular signature.

### Tumor antigen heterogeneity

One of the barriers to the effectiveness of cell therapy against solid tumors is antigen heterogeneity, which impairs the detection of cancer cells by T cells and reduces the impacts of CART therapy. Since the most useful targets for CAR engineering are tumor-associated antigens (TAA), the diverse expression of TAA by different types of tumor cells is a major barrier. Furthermore, different levels of antigen expression at various tumor sites may impair the function of CAR T cells at the tumor location because malignant cell antigen diversity makes it difficult to identify tumor cell-specific antigens [108].

So far, various methods have been used to support the targeting of multiple TAAs by identified CAR T cells, including the co-expression of several CARs on a single T cell, programmable CAR expression, possibility of temporary adjustment of target antigens, exploiting of various CAR T cells, the expression of each chimeric receptor relative to a specific antigen, and expression of a chimeric receptor including two or more antigen recognition domains, which in turn leads to the multiple antigens identifying through the individual receptor [109]. On the other hand, targeting cancer stem cells that are closely related to tumor heterogeneity is one of the methods to eliminate tumor heterogeneity. For example, CD133 is a tumor stem cell marker that is overexpressed in many solid tumors and is now considered a target tumor marker for CAR-T cells [13].

### Trafficking and infiltration into tumor tissue

**Fig. 5:**
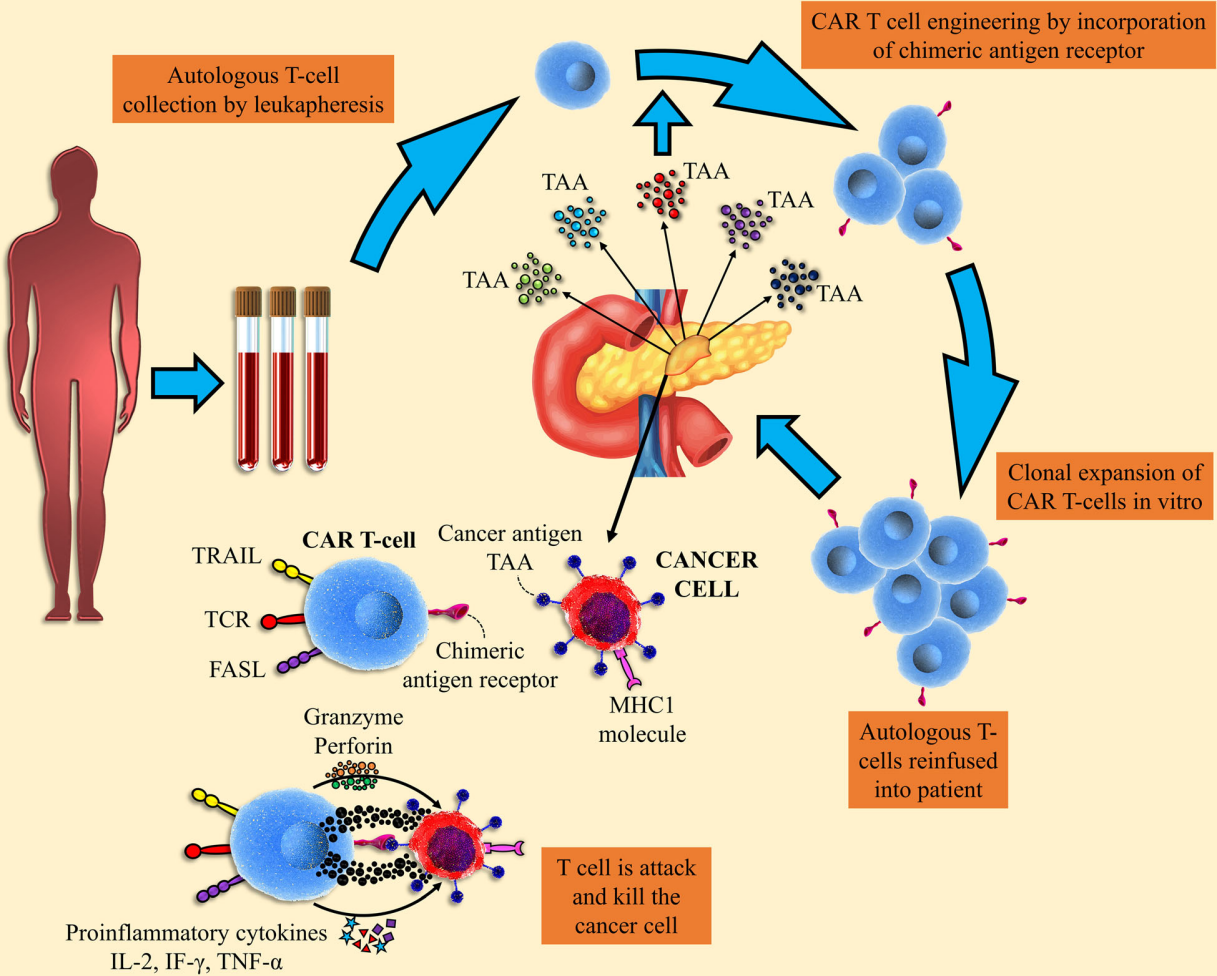
Isolation of CAR T cells and its interaction with tumor-associated antigens (TAA) in solid tumors. T cells are collected from patients’ peripheral blood via leukophores and are designed to express chimeric antigen receptors to tumor-specific antigens. These cells proliferate before being re-injected. After injection, autologous CAR-engineered T cells detect TAA and binds to its corresponding ligand, leading to the secretion of cytokines and the interaction of some apoptosis-related ligands, which ultimately leads to the destruction of tumor cells

CAR-T cell therapy is more limited in solid tumors than in hematological tumors, because CAR-T cells return to the bloodstream and lymphatic system, so they have more contact with blood tumor cells, whereas in solid tumors, CAR-T cells may not be able to penetrate tumor tissue through the vascular endothelium [110]. The presence of a set of mechanisms in tumor tissue reduces the secretion of vascular-related factors. For example, overexpression of endothelin B receptors in cancer tissues downregulates the ICAM-1 level and thus prevents T cells escape from the blood vessels [111]. On the other hand, the migration of CAR-T cells in solid tumors depends on the adjustment of chemokines such as ligand-11 and 12 chemokines [112]. However, these chemokines are less expressed in tumor tissue. In summary, due to the lack of expression of chemokines involved in the penetration of T cells into tumor tissues, as well as the presence of dense fibrotic matrix in solid tumors, CAR’s ability to migrate and invade tumor cells is reduced [71]. Identifying solid tumors requires cells to transition from the blood into the cancer site, and various abnormalities develop such that T cell infiltration is roughly blocked [113, 114]. It has been proposed that in sites where the tumor is restricted, regional administration of CAR T cells is more effective than their systemic administration. Intracranial transport has been exposed to be safe and to have an acceptable anti-cancer effect in glioblastoma [115], and intra-pleural transport of CAR T cells was more effective than their systemic administration in human pleural malignancy preclinical researches [116]. Advance understanding of the process that improves or excludes T cell access to tumors are expected to shape opportunities to augment CAR T cell trafficking [117], either by further genetic variances of T cells or by composed use of CAR T cells in association with oncolytic viruses or other approaches, which finally enhance inflammatory response at the tumor location [118]. The CAR T cells can be modified to express chemokine-specific receptors, in particular, CCR2 and CCR4, certainly overexpressed by tumors, supporting their efficient contact with tumor cells (Fig. 5) [119]. Rather than routine engineering T cells to the special cancers chemokine profile, a more acceptable method is to persuade tumors to release chemokines, which CAR T cells are previously responsive to. One type of oncolytic virus has been utilized to convey the chemokine CCL5 to the tumor cells. CAR-T cells commonly express receptors RANTES receptors, such as CCR1, CCR3, and CCR5, and combined use of CCL5-expressing oncolytic virus with engineered CAR T cells powerfully promoted the viability and tumor clearance in some of the preclinical studies [118, 120].

### Immunosuppressive tumor microenvironment

Another important challenge for the effective targeting of solid tumors with CAR T cell therapies is the immunosuppressive tumor milieu. Unlike many hematological malignancies that lack local immune suppression pathways, solid tumors can be strongly infiltrated by different cell types that support tumor growth, angiogenesis, and metastasis [121]. Regulatory T cells (Tregs), myeloid-derived suppressor cells (MDSCs), and M2 tumor-associated macrophages (TAMs) are the most prominent types of immune suppressor cells in the tumor environment [122, 123]. In addition to tumor cells, these cells facilitate tumor growth and proliferation by producing growth factors, local cytokines, and chemokines in solid tumors, including VEGF and IL-4, IL-10, and TGFβ. immune checkpoint molecules such as CTLA-4 and PD-1 also reduce antitumor immunity [120, 124]. In general, a tumor microenvironment with multiple cells and inhibitory agents can restrict the influence of CAR T cell treatment. A large number of studies have focused on enhancing CAR T cell function by modifying their metabolic profiles to improve cell activity in hostile environments. Usually, tumors are often described by a high degree of adenosine and reactive oxygen species (ROS), disrupting T cell responses (Fig. 5) [125, 126]. Likewise, tumors demonstrate promoted levels of extracellular potassium that prominently weaken TCR-driven Akt-mTOR phosphorylation and subsequent effector activity. T cell engineering aims to increase the expression of potassium channel to prepare greater potassium efflux successfully undoes this type of suppression and boost T cell function within the TME [127]. Researches have demonstrated that in the TME, the defeating of the immunosuppressive cells is routinely necessary to the high-level efficacy of CAR T cells. Using suppressor antibodies in association with genetic manipulation with the aim of depletion of regulatory T cells (Tregs), as well as myeloid-derived suppressor cells (MDSCs), leads to the promotion of T cell therapy efficacy in animal models (Fig. 5) [128, 129]. On the other hand, cancer-associated fibroblasts (CAFs) that include the most common types of TME cells and express fibroblast activation protein (FAP) in a high degree has a crucial role in shaping the immunosuppressive microenvironment and releasing of the ECM proteins to attenuate T cell penetration. Interestingly, applying the FAP-specific CAR T for reduction of CAF cell activity or engineering novel types of the CAR T cells aiming to secrete ECM-degrading enzymes can remarkably increase their potential to traffic and lyse tumors [130]. Otherwise, CAR T cell manipulation to secrete the pro-inflammatory cytokine IL-12 may modify the TME and finally enhance macrophage recruitment and functions [131]. Numerous groups have tried to improve CAR T cell activity by the combined use of the ACT with TME modulators. A hopeful therapeutic method that has exposed acceptable efficacy in tumors is the use of the checkpoint inhibitors, which target the PD-1/PD-L1 or CTLA-4 pathways (Fig. 5) [132, 133]; in this case, checkpoint blockade is ameliorated following improving the preparation of tumor-specific T cells and may rationally be composed with the adoptive transmission of CAR T cells, while the risk of toxicity may be improved in normal calls. On the other hand, particular CAR T cells have engineered to release anti-PD-L1 antibodies to PD-1 and LAG3 suppressing through CRISPR [134, 135]. While anti-CTLA-4 antibodies are able to increase endogenous T cell reactions to the cancers, the related mechanism by which they can promote CAR T cell responses is unknown. Furthermore, anti-CTLA-4 antibodies can trigger an immune reaction in a cell-extrinsic manner after diminishing of CTLA-4+ Treg cells, which in turn may likely assistance CAR T cells [136].

## Future directions and conclusion

Development in CAR T cell therapy is a promising therapeutic option for patients with advanced malignancies, in particular, blood disorders. The progression of CAR T cells reflects a merging of perceptions from various scientific fields; however, success has so far been restricted to the B cell abnormalities. Progression of this therapeutic method to solid tumors will demand the improvement of plans based on the recognition of the impediments posed by TME in association with tumor heterogeneity, which is emerging from intricate logical tools and high-importance models. These approaches will take benefit from our ability to establish genetically modified T cells to support novel desired activities, aiding them to target solid tumor cells and persist and act in hostile circumstances.

## Data Availability

Not applicable
